# Obsessive-compulsive disorder and attention-deficit/hyperactivity disorder: distinct associations with DNA methylation and genetic variation

**DOI:** 10.1186/s11689-020-09324-3

**Published:** 2020-08-16

**Authors:** Sarah J. Goodman, Christie L. Burton, Darci T. Butcher, Michelle T. Siu, Mathieu Lemire, Eric Chater-Diehl, Andrei L. Turinsky, Michael Brudno, Noam Soreni, David Rosenberg, Kate D. Fitzgerald, Gregory L. Hanna, Evdokia Anagnostou, Paul D. Arnold, Jennifer Crosbie, Russell Schachar, Rosanna Weksberg

**Affiliations:** 1 Genetics and Genome Biology, SickKids Hospital, Toronto, ON Canada; 2 Neurosciences and Mental Health Program, SickKids Hospital, Toronto, ON Canada; 3 Department of Pathology and Molecular Medicine, McMaster University, Hamilton, ON Canada; 4 Biochemical Genetics Laboratory, Alberta Children’s Hospital, Calgary, AB Canada; 5 Centre for Computational Medicine, SickKids Hospital, Toronto, ON Canada; 6 Department of Computer Science, University of Toronto, Toronto, ON Canada; 7 Department of Psychiatry and Behavioural Neurosciences, McMaster University, Hamilton, ON Canada; 8 Department of Psychiatry and Behavioral Neurosciences, Wayne State University, Detroit, MI USA; 9 Department of Psychiatry, University of Michigan, Ann Arbor, MI USA; 10 Holland Bloorview Kids Rehabilitation Hospital Toronto, Toronto, ON Canada; 11 Institute of Medical Science, University of Toronto, Toronto, ON Canada; 12 Mathison Centre for Mental Health Research and Education, University of Calgary, Calgary, AB Canada; 13 Departments of Psychiatry and Medical Genetics, Hotchkiss Brain Institute, Cumming School of Medicine, University of Calgary, Calgary, AB Canada; 14 Department of Psychiatry, University of Toronto, Toronto, ON Canada; 15 Division of Clinical and Metabolic Genetics, SickKids Hospital, Toronto, ON Canada; 16 Department of Molecular Genetics, University of Toronto, Toronto, ON Canada; 17 Department of Paediatrics, University of Toronto, Toronto, ON Canada

**Keywords:** DNA methylation, Epigenetics, OCD, ADHD, Biomarker

## Abstract

**Background:**

A growing body of research has demonstrated associations between specific neurodevelopmental disorders and variation in DNA methylation (DNAm), implicating this molecular mark as a possible contributor to the molecular etiology of these disorders and/or as a novel disease biomarker. Furthermore, genetic risk variants of neurodevelopmental disorders have been found to be enriched at loci associated with DNAm patterns, referred to as methylation quantitative trait loci (mQTLs).

**Methods:**

We conducted two epigenome-wide association studies in individuals with attention-deficit/hyperactivity disorder (ADHD) or obsessive-compulsive disorder (OCD) (aged 4–18 years) using DNA extracted from saliva. DNAm data generated on the Illumina Human Methylation 450 K array were used to examine the interaction between genetic variation and DNAm patterns associated with these disorders.

**Results:**

Using linear regression followed by principal component analysis, individuals with the most endorsed symptoms of ADHD or OCD were found to have significantly more distinct DNAm patterns from controls, as compared to all cases. This suggested that the phenotypic heterogeneity of these disorders is reflected in altered DNAm at specific sites. Further investigations of the DNAm sites associated with each disorder revealed that despite little overlap of these DNAm sites across the two disorders, both disorders were significantly enriched for mQTLs within our sample.

**Conclusions:**

Our DNAm data provide insights into the regulatory changes associated with genetic variation, highlighting their potential utility both in directing GWAS and in elucidating the pathophysiology of neurodevelopmental disorders.

## Background

Attention-deficit/hyperactivity disorder (ADHD) and obsessive-compulsive disorder (OCD) are common, heterogeneous disorders that can co-occur or occur with other neurodevelopmental disorders (NDDs), including autism spectrum disorder (ASD) and Tourette syndrome (TS) [1–3]. Elucidating the etiologies and pathophysiologies of these disorders has proven challenging as they have historically been classified based on varying clinical profiles, rather than underlying biology [4].

ADHD is characterized by inattention, hyperactivity, and impulsivity mostly in childhood but it can persist into adolescence and adulthood [5–7]. ADHD affects approximately 5% of children and adolescents, and 2.5% of adults [8]. Core features of OCD consist of recurrent and unwanted thoughts, urges, and repetitive behaviors or mental acts performed to reduce anxiety or a sense of dread [9]. These behaviors and thoughts can impair social and occupational functioning in individuals with OCD [9]. The estimated prevalence of OCD in childhood and adult populations is similar, approximately 1–3% [10, 11].

Both ADHD and OCD have been the focus of considerable genetic research, including a small number of genome-wide association studies (GWAS), given their relative heritability estimates of 70–80% and 40–65%, respectively [12–16]. Both disorders have been found to be polygenic in nature, with many common single nucleotide polymorphisms (SNPs) each conferring small risks [17–22]. However, there has been a notable lack of reproducible GWAS findings, which may be attributed to lack of statistical power but also heterogeneity in the disorders [23–25]. Accounting for this heterogeneity by examining symptom severity rather than diagnostic categories may help increase statistical power since individuals with more severe symptoms plausibly have a larger genetic load. The hypothesis that the manifestation of each disorder represents extremes of a quantitative trait may explain the heterogeneity of these disorders and the rarity of replicable risk variants despite strong heritability [26, 27].

In addition to genetics, epigenetic factors might mediate the expression of ADHD and OCD. Epigenetics refers to heritable changes to the chromatin state that are not due to changes in DNA sequence, such as those accompanying cellular reprogramming [28, 29]. DNA methylation (DNAm), the most commonly studied human epigenetic mark, can reflect both genetic and environmental influences in a quantitative and often stable manner [30, 31]. To that end, DNAm states in 20–80% of CpGs in the genome are thought to associated with genetic variation to some extent [32–35], and inter-individual variation of DNAm in a single CpG is best predicted by an interaction between genetics and environment [30]. Research in ADHD has identified numerous environmental risk factors including birth weight, early-life maltreatment, lead exposure, and maternal smoking during pregnancy [18, 36–38]. In contrast, there is currently a lack of convincing evidence for reproducible associations between OCD and environmental factors [14, 39].

In ADHD and OCD, a small number of DNAm studies, including candidate analyses and epigenome-wide association studies (EWAS), have been published. Most notably, a recent EWAS of ADHD performed on DNAm measured in whole blood, found a large degree of heterogeneity across three ADHD cohorts, with no differentially methylated sites replicating in the meta-analysis [40]. Additional ADHD EWAS have been performed in cord blood and saliva, the latter identifying differentially methylated sites in *VIPR2*, a gene encoding a protein that plays a role in circadian rhythm [41, 42]. Research into DNAm patterns associated with OCD is more limited. One epigenetic OCD analysis reported DNAm associations proximal to genes involved in actin binding, cell adhesion and transcriptional regulation [43]. Targeted analyses have also implicated *BDNF* and *OXTR* DNAm in OCD [44, 45].

While research into the epigenetic patterns underlying ADHD and OCD is still relatively nascent, EWAS of schizophrenia have provided strong evidence that epigenetic research can focus and strengthen genetic research [46–48]. An integrated analysis of genetics and DNAm in schizophrenia found that (1) differentially methylated sites associated with a diagnosis of schizophrenia replicated across independent cohorts, (2) differentially methylated sites corresponded to known schizophrenia GWAS loci, and (3) GWAS loci were enriched for methylation quantitative risk loci (mQTLs) [46, 49].

Here, we undertook a novel approach of incorporating genetics, phenotype, and epigenetics to identify DNAm correlates of ADHD and OCD. We hypothesized that disorder heterogeneity would be reflected in DNAm patterns and categorized individuals by their clinical profile to aid in identifying differentially methylated sites. We ran linear models of DNAm in ADHD or OCD cases vs. controls and compared the results to the same analyses run on a subset of ADHD or OCD cases selected based on severity or number of symptoms. We then assessed whether restricting heterogeneity of the phenotype led to a stronger epigenetic signal. We also tested the disorder-associated CpGs for their relatedness to nearby genetic variation, i.e., mQTLs, and finally, assessed how these mQTLs were positioned in independent GWAS findings. We found that DNAm is a better discriminator of more symptomatic cases of ADHD and OCD than the heterogeneous, full cohorts of cases, as compared to controls. As well, CpG sites differentially methylated between cases and controls, in both ADHD and OCD analyses, were enriched for mQTL associations.

## Methods and materials

### Participants

Information on participants can be found in Table 1.

**Table 1:** Sample sizes and demographics

Cohort	*n*	study	Age years (med.)	Sex (% F)	Array batch
control	54	Michigan (*n* = 19); TAG (*n* = 35)	4–19 (12)	27%	A (10); B (9); C (35)
OCD	59	POND (*n* = 33); Michigan (*n* = 26)	7–13 (9)	44%	A (16); B (15); C (28)
ADHD	22	POND (*n* = 22)	7–17 (8)	56%	all A

Participants for this study were collected from three unique cohorts: (1) Patients with OCD and matched controls were recruited from the Department of Psychiatry at the University of Michigan and surrounding community. The lifetime and current severity of OCD was assessed in patients with a modified version of the Children’s Yale-Brown Obsessive Compulsive Disorder Scale (CY-BOCS), with patients and their parents providing item scores retrospectively for the most severe episode of OCD and item scores for current severity. (2) Patients with ADHD or OCD were recruited through the Province of Ontario Neurodevelopmental Disorders Network (POND) from The Hospital for Sick Children (SickKids Hospital; Toronto), Holland Bloorview Kids Rehabilitation Hospital (Toronto), McMaster Children’s Hospital (Hamilton), or Lawson Health Research Institute (London). Participants were recruited if they had a primary clinical diagnosis of ADHD or OCD, sufficient English comprehension to complete required testing, and no contraindications for MRI. Diagnoses were established using the Parent Interview for Child Symptoms for ADHD and the CY-BOCS for OCD. (3) Age-, sex-, and tissue-matched control samples were measured in individuals recruited at the Ontario Science Centre in Toronto as part of the Spit for Science study [details published elsewhere (Crosbie et al.)] [50]. In total, 17,262 children and adolescents between 6 and 17 years were recruited. Participants were excluded if they had reported receiving a diagnosis of any mental illness from a physician or mental health professional in an electronic questionnaire (community diagnosis). Parents of children younger than 13 years filled out the questionnaires on their child’s behalf (referred to as “parental respondents”). Individuals age 15 and older completed the questionnaires for themselves, while those between the ages of 13 and 15 responded either for themselves or had parents fill out the questionnaire. Our previous work established that the incidence rates of self-reported diagnoses of NDDs in this community sample were comparable to population prevalence as are typically reported (see OMIM 20985; OMIM 143465; OMIM 164230) [50, 51]. Approval from research ethics boards was obtained at all participating institutions. For all patients, parental consent was obtained for children between 6 and 12 years of age. Individuals who were 13 years and older provided their own consent in addition to parental consent.

### Sample selection of ADHD and OCD and symptom characterization

Samples selected from the three cohorts for the analysis presented here met a number of criteria. Firstly, we imposed a limit of one case, ADHD or OCD, per family. Cases could have symptoms of other disorders (e.g., ADHD case with some OCD symptoms) but not comorbid diagnoses at the time of data collection (e.g., child with ADHD and ASD). Individuals were required to be European Caucasian ancestry due to the strong association between DNAm and ethnicity or heritage [52, 53]. Detailed medication history was collected, and anyone with a history of seizure medication (e.g., valproic acid) was excluded due to known effects on one-carbon metabolism, the biochemical pathway in which methyl donors are produced. Following case selection, a similar number of age- and ancestry-matched controls were chosen.

We selected ADHD and OCD cases based on cutoffs on the SWAN and CY-BOCS, respectively [54–57]. For the ADHD sample, a threshold of ≥ 6 symptoms based on the SWAN was used which reflects the DSM-5 criteria [9].

For the OCD sample, a threshold of CY-BOCS total score ≥ 18 was used. We selected a slightly more conservative cutoff than that suggested for the CY-BOCS “moderate” symptom severity range. Twenty of 59 OCD samples did not have CY-BOCS scores and as such were excluded from analysis of the “more symptomatic” OCD subset. However, as there were no a priori requirements of disorder severity in the full OCD sample analysis, these individuals were included there.

### DNAm data generation and preprocessing

Saliva was collected using Oragene OG-500 (DNA Genotek, Ottawa, ON) collection kits and stored at room temperature as per manufacturer’s instructions. DNA was extracted from saliva for all cases and controls using standard techniques. Extracted DNA was sodium bisulfite converted using the Qiagen EZ DNA Methylation kit (Qiagen, Valencia, CA), according to the manufacturer’s protocol. All DNA samples were processed according to the manufacturer’s protocol for DNAm analysis using the Illumina Human Methylation 450 K (450 K array) at The Centre for Applied Genomics (SickKids). The distribution of the samples on the arrays was randomized for all cases and controls and for age and sex.

Raw data (IDAT files) underwent pre-processing quality control and normalization prior to analysis, using the R package minfi [58]. Low quality probes were removed, as measured by the detection *p* value, as well as probes located on sex chromosomes, cross-reactive probes, SNP probes, and probes targeting CpG sites within 5 bp of an SNP with a minor allele frequency > 1%. Background signal subtraction and control normalization were then performed using the methods designed for the Illumina Genome Studio software. The final output consisted of 426,551 methylation values for each sample (Beta [β] values) ranging from 0 to 1, corresponding to the percent methylated probes measured at each CpG.

Prior to statistical analysis, buccal epithelial cell (BEC) and blood cell proportions were estimated from the methylation data using methods similar to those described in Houseman et al. for blood samples and Smith et al. for saliva samples [59, 60]. We used isolated cell types (GEO GSE46573, GEO GSE35069) as reference methylomes to identify CpGs differentially methylated by cell type and then predicted the cellular composition of each saliva sample [61, 62].

### Genotyping data generation and preprocessing

The samples were genotyped as part of different genotyping projects, on a variety of genotyping arrays: Illumina HumanCoreExome, PsychArray, Omni2.5, and Affymetrix6.0. Samples for each array type were processed separately, using the same pipeline described below. Data for each sample was extracted from imputed data, combined and analyzed. Samples were excluded for the following technical reasons: if (1) their call rate was below 97% (2), if they were found to be outliers with respect to heterozygosity, where outliers are defined as a value at a distance greater than 6 times the interquartile range from the closest quartile, and (3) if the sex predicted from the genotypes differed from the reported sex. SNPs were excluded if (1) their call rate was below 97%, (2) deviated from the rules of Hardy-Weinberg equilibrium at an FDR < 1%, based on a set of homogeneous samples in terms of ancestry, and (3) were found to be duplicates of other SNPs, based on position and alleles, in which case the one with the highest call rate was retained. These statistics were computed using plink v1.90 [63].

Imputation was performed separately for each project, using Beagle v4.1 and companion program conform-gt with default values. A/T and C/G genotyped SNPs were removed prior to imputation. Data from phase 3, version 5 of the 1000 Genomes project, downloaded from http://bochet.gcc.biostat.washington.edu/beagle/1000_Genomes_phase3_v5a/b37.vcf/, was used as reference.

Principal components (PCs) were calculated from a set of autosomal, bi-allelic ancestry informative markers (AIM), calculated from samples from phase 3 of the 1000 Genomes project. We first pruned SNPs for linkage disequilibrium (*r*^2^ < 0.2 in 1500 kbp windows). Then, for each continental population, the top 1% SNPs with largest frequency differences between that population and all others were retained. We ignored SNPs in the MHC regions: chr8 7,000,000–13000000 [hg19] (8p23 inversion) and chr6 25,000,000-34,000,000.

Samples’ AIMs were extracted from the imputed data sets, as long as their imputation quality was AR2 > 0.8. Hard genotype calls were used. To identify outliers with respect to ancestry, i.e., non-Caucasian samples, data from samples were combined with data from the 1000 Genomes project (Supplementary Figure 1). PCs were calculated using plink v1.90, and outliers (as defined above) were identified from each of the top 3 principal components. Once ancestry outliers were removed, PCs were recomputed without 1000 Genomes samples and used as covariates in downstream statistical analyses.

### GWAS datasets

Additional datasets used for investigating the relationship between genotype and ADHD or OCD diagnosis at SNPS of interest were attained through the Psychiatric Genetics Consortium (https://www.med.unc.edu/pgc/results-and-downloads). Summary statistics from Demontis et al. and IOCDF-GC and OCGAS were downloaded to assess genotype-phenotype correlations in independent samples of European ancestry [19, 22]. The ADHD GWAS was performed on 19,099 individuals with ADHD (and 34,194 matched controls from the European Caucasian subset), and the OCD GWAS was performed on 2688 individuals with OCD and 7037 matched controls [19, 22].

### Statistical analyses

**Fig. 1:**
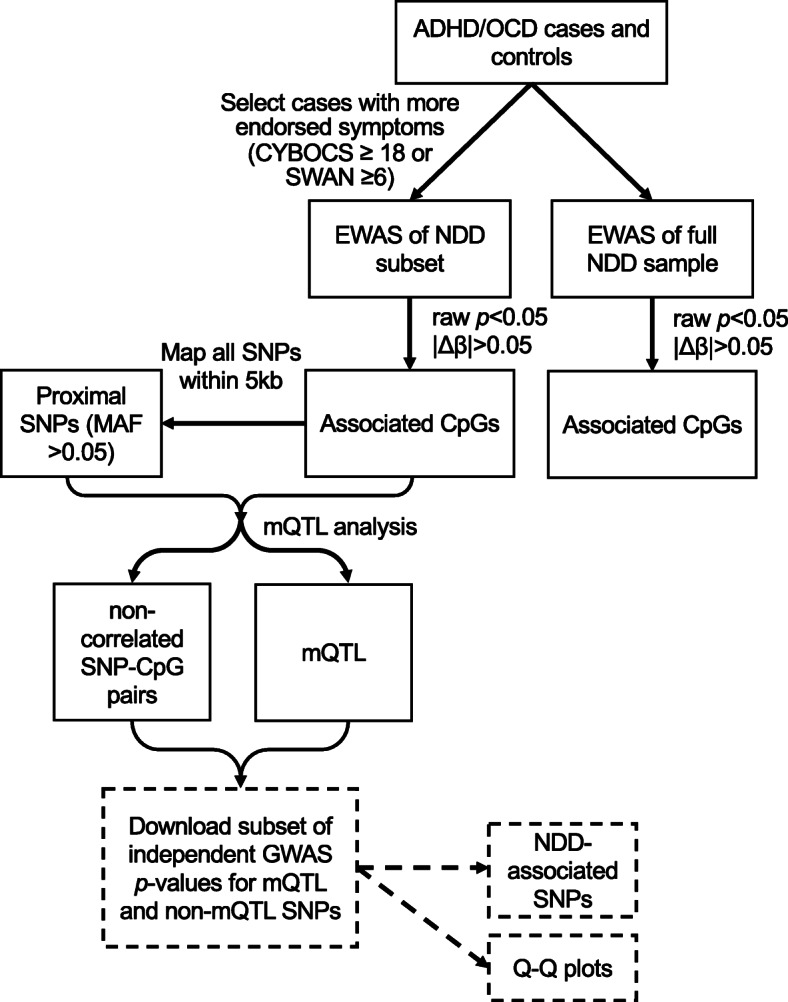
Pipeline of statistical analysis. Black boxes and arrows indicate that the analyses were performed on our ADHD and OCD cases versus controls. Sample sizes for each comparison can be found in Table 2. Dashed boxes and arrows indicate analyses that were performed on independent samples and previously published (Demontis et al. 2017; IOCDF-GC and OCGAS 2017); summary statistics downloaded from the Psychiatric Genetic Consortium were used

Analysis pipeline is summarized in Fig. 1.

Genome-wide DNAm analyses were performed for each two-group comparison using the Limma package, which runs a linear regression on each CpG. Sex, age, and estimated buccal proportion were included as covariates, as well as batch, where appropriate; as all ADHD samples were run in a single batch, only controls from the same batch were included in these comparisons. While both buccal cell and granulocyte proportions were estimated, these measures were strongly inversely proportional and as such, the granulocyte measure was not included as a covariate. CpGs reported as significantly associated with ADHD or OCD were required to have a nominal *p* value < 0.05 and an absolute Δβ > 5%. Δβ is calculated as the difference in mean DNAm (β) between groups. While Benjamini-Hochberg correction for multiple testing was applied, it was not reported as no sites met a threshold of *q* value < 0.05.

Principal component analysis (PCA) was performed on mean-centered data using Qlucore Omics Explorer [QOE, www.qlucore.com] for visualization of case-control clustering. Silhouette scores were calculated using beta values and Manhattan distances for clustering.

mQTL identification was performed by first identifying all SNPs within 5 kb of any CpG significantly associated with ADHD or OCD in the more symptomatic samples subsets. SNPs with < 5% minor allele frequency (MAF) in our sample (either ADHD and appropriate controls or OCD and appropriate controls, depending on the SNP) were removed. Alleles at each SNP were coded as “0”, “1”, and “2”, and a Spearman correlation was run at each SNP-CpG pair. MQTLs were identified as SNP-CpG pairs with a Benjamini-Hochberg corrected correlation *p* value < 0.05 [52].

To test if the number of OCD- or ADHD-associated CpGs associated with mQTLs were significantly enriched compared to background CpGs (i.e., CpGs assayed on the EPIC array), we employed repeated random sampling. For each disorder, we randomly sampled a set of CpGs equal to the number of disorder-associated CpGs, 1000 times. For each iteration, the same methods used above for mQTL identification were applied: first, mapping all variable SNPs within 5 kb of each CpG, running correlations, and finally, correcting *p* values for false discovery rate. The output of each iteration was the sum of CpGs associated with at least one SNP (mQTL); combined, these 1000 sums were used to generate a random null distribution.

Finally, a logistic regression using disorder status as the outcome was run on each SNP that was significantly associated with an NDD-associated CpG (3283 SNPs correlated with ADHD-associated CpGs and 1150 SNPs correlated with OCD-associated CpGs), using the R package *snpStats* [64]. Principal components 1 and 2 calculated from the full genotyping array data were included as covariates to account for population substructure. Disorder-associated SNPs met a Benjamini-Hochberg corrected *p* value < 0.05.

Summary statistics from independent GWAS were downloaded for two sets of SNPs identified using the prior ADHD and OCD mQTL analyses. First, all SNPs identified as mQTLs and second, all SNPs tested in mQTL analyses but not significantly associated with DNA methylation, were assessed for their association to either ADHD or OCD, as reported in each GWAS [19, 22]. Q-Q plots and genomic inflation factors (*λ*) were generated from the GWAS *p* values of these subsets to assess if SNPs proximal to CpGs associated with a disorder were more likely to be associated with the disorders themselves, as indicated by positive skewing of observed *p* values and larger *λ* values, respectively.

## Results

### DNAm better distinguishes more symptomatic cases of ADHD and OCD from controls, as compared to more heterogeneous, full case sets

DNAm profiles of all ADHD and OCD samples (*n* = 22, *n* = 59, respectively) were compared with age-, and tissue-matched controls (*n* = 35, *n* = 54, respectively) at 426,551 sites using linear regression and covarying for sex, buccal cell proportion, and age. Buccal composition was estimated using DNAm and was included as a covariate despite no significant differences between cases and controls (data not shown), as cellular heterogeneity is strongly associated with DNAm. Batch was also included as a covariate for analysis of OCD samples and the corresponding controls as these were run in three batches, with equal numbers of cases and controls in each batch. No sites were identified as significantly associated with ADHD or OCD after Benjamini-Hochberg correction for multiple testing (all *q* > 0.05). As such, we set the criteria for significance to a nominal *p* < 0.05 and |Δβ| > 5% to identify sites likely to be true associations while remaining cognizant of the increased risk of false positives. At this threshold, 188 CpG sites were associated with ADHD, and 82 CpGs were associated with OCD (Supplementary Table 1). Seven sites were associated with both ADHD and OCD and mapped to the following genes: *DNAJC15* (2 CpGs), *C13orf39*, *DLGAP2*, and *PRDM9*; two CpGs were intergenic.

As both ADHD and OCD are heterogeneous disorders, we repeated our analyses on subsets of cases that included only individuals who were more symptomatic, as determined by ≥ 6 SWAN symptoms for ADHD or CY-BOCS total score ≥ 18 for OCD (*n* = 15; *n* = 28, respectively). Of note, a higher CY-BOCS corresponds to more severe OCD symptoms, while a higher SWAN score is indicative of more ADHD symptoms, i.e., more behaviors reflecting inattention, hyperactivity, or impulsiveness. Although there were still no significant differences in buccal proportion found between cases and controls (both *p* values> 0.05), differences in the distribution of buccal proportion in the subset of ADHD and controls were apparent (Supplementary Figure 2). To better balance buccal proportion in ADHD cases and controls, controls were stratified by cell proportion, and eight samples were removed (remaining control samples *n* = 27). As well, controls were significantly older than the ADHD subset; this was the only comparison for which age differed significantly between cases and controls, and age was used as a covariate in all statistical models (Supplementary Figure 3).

**Fig. 2:**
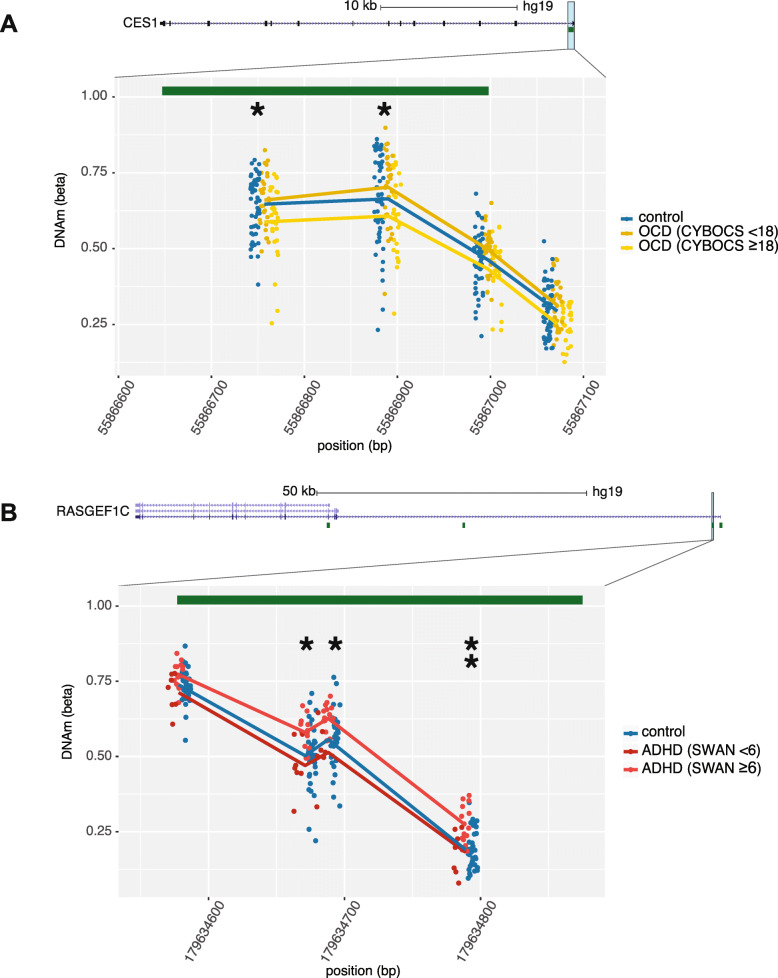
Differential methylation found in subset ADHD and OCD cohorts. CpG sites denoted by asterisks were differentially methylated in (**a**) *CES1* in the subset of OCD (determined by CY-BOCS ≥ 18, *n* = 28) and (**b**) *RASGEF1C* in the subset of ADHD (SWAN symptoms ≥ 6, *n* = 15), as compared to controls. CpG sites denoted by two asterisks remained significant in full ADHD cohort. Lines represent mean methylation at each CpG in (1) controls, (2) the subset of more symptomatic cases, and (3) remaining cases. Green bars represent CpG islands

Linear models, identical to those run on the full cohorts, were then applied to the more optimally matched groups, and disorder-associated CpGs were identified using the same criteria, i.e., nominal *p* < 0.05 and |Δβ| > 5%. In both ADHD and OCD, a greater number of sites were associated with the more symptomatic subsets than the full cohorts, which is likely due to the decreased heterogeneity of these subsets (299 ADHD-associated CpGs; 137 OCD-associated CpGs; Supplementary Table 2). Additionally, many significant CpGs mapped to the same gene, suggestive of differentially methylated regions (DMRs); these included *POUF6* (6 CpGs), *PRDM8* (4 CpGs), *SNRPN* (4 CpGs), and *RASGEF1C* (3 CpGs) associated with the ADHD subset, and *NINJ2* (5 CpGs), *PRKG1* (4 CpGs) and *CES1* (2 CpGs) associated with the OCD subset (example DMRs shown in Fig. 2). The overlap in CpGs associated with both the full cohort and more symptomatic subset was greater than expected by chance in both ADHD and OCD (103 CpGs and 35 CpGs, respectively), as determined by random resampling 1000 times (all *p* < 0.0001; Table 2).

**Table 2:** Statistical comparisons

comparison	criteria for subsetting	cases (*n*)	controls (*n*)	covariates	# CpGs	# overlapping CpGs
ADHD vs. controls	NA	22	35	sex, age, %BEC	188	103
	SWAN ≥ 6	15	27		299	
OCD vs. controls	NA	59	54	sex, age, %BEC, batch	82	35
	CYBOCS ≥ 18	28	54		137	

**Fig. 3:**
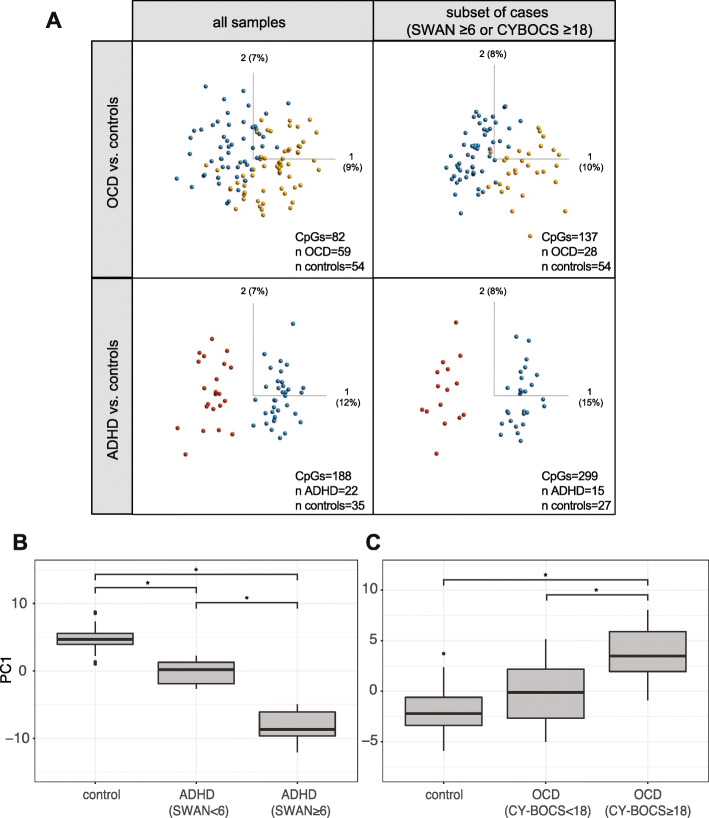
PCA plots of NDD-associated CpGs and relative PC1 scores in controls, “less symptomatic”, and “more symptomatic” individuals with ADHD and OCD. **a** Samples sizes and number of CpGs input into PCA shown in bottom, right-hand corner of each facet. **b** PC1 scores of PCA run on 299 CpGs differentially methylated between controls and the more symptomatic ADHD subset, with “less symptomatic” samples included in PCA (*n* controls = 27, *n* ADHD less symptomatic = 7, *n* ADHD more symptomatic = 15). **c** PC1 scores of PCA run on 137 CpGs differentially methylated between controls and the more symptomatic OCD subset with “less symptomatic” samples included in PCA (*n* controls = 54, *n* OCD less symptomatic = 11*, n* OCD more symptomatic = 28, *n* = 20 removed due to missing CY-BOCS scores). Comparisons were performed using ANOVA and marked by asterisks if significant (Tukey *p* values < 0.05)

To assess how well the cases clustered separately from controls, i.e., how unique their methylation profiles were at the disorder-associated sites, we ran PCA on all four sets of disorder-associated sites (ADHD full cohort, ADHD more symptomatic subset, OCD full cohort, OCD more symptomatic subset) on the samples from which they were derived (Fig. 3a). In both ADHD and OCD, the more symptomatic subsets of cases clustered farther from controls, as compared to the full cohort. As well, PC1, which separated cases from controls in all comparisons, accounted for a larger proportion of the total variation in the PCA performed on the subsets, as compared to the full cohort (PC1 ADHD subset = 15%, PC1 ADHD full = 12%; PC1 OCD subset = 10%, PC1 OCD full = 9%).

To quantify the differences observed in the PCA plots, silhouette widths, a measure of the average distance between clusters, were compared using the beta values and Manhattan distance (Supplementary Figure 4). In both ADHD and OCD, average silhouette widths increased in the subsets containing only more symptomatic cases compared to controls. As well, among the samples included in both the full analysis and the subset silhouette widths were significantly greater (Wilcoxin signed-rank *p* values< 0.05). We then re-introduced the less symptomatic samples (ADHD *n* = 7, OCD *n* = 11) into the PCAs of 299 ADHD subset-associated CpGs and 137 OCD subset-associated CpGs and found that PC1 scores of these less symptomatic samples fell between more symptomatic cases and controls (Fig. 3b, c). Overall, these visualizations and quantitative tests all suggest that more symptomatic cases of ADHD and OCD demonstrate greater DNAm differences from controls.

Finally, to assess whether the difference in sample selection or CpG set was responsible for the greater separation between cases and controls in the more symptomatic subset, we performed PCA on the complete sample set using CpGs identified from the more symptomatic cohort. As well, we performed PCA on the more symptomatic sample set using CpGs identified from the complete cohort, in both ADHD and OCD samples. The more symptomatic samples remained more distantly clustered from controls, as compared to the complete cohort of cases (Supplementary Figure 5). Irrespective of CpG set, the methylation patterns of the more symptomatic individuals were more distinct from the control samples.

### Disorder-associated CpGs were enriched for mQTLs

Next, we assessed disorder-associated CpGs for underlying mQTLs, given the common relationship between genetic DNAm variation, especially in NDD related loci. We filtered for variable SNPs within a 5-kb window of the two sets of disorder-associated CpGs identified using the more symptomatic subsets, as they had better separation from controls. We then ran Pearson correlations between genotypes, coded numerically, and DNAm to identify mQTLs. Of the 299 CpGs associated with ADHD, 263 were tested with SNPs within 5 kb, and 88% of those (232) were significantly associated with at least one SNP at an FDR-corrected *p* value < 0.05. A total of 6433 SNP-CpG pairs were tested, as one CpG could be tested against multiple SNPs within 5 kb, and 3283 were identified as mQTLs.

**Fig. 4:**
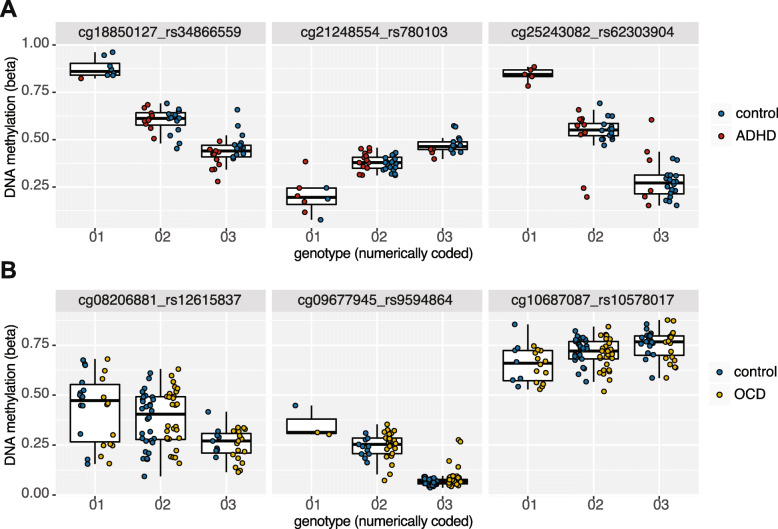
Boxplots of sample mQTLs identified in (**a**) ADHD cases and controls (*n* = 42) and (**b**) OCD cases and controls (*n* = 82). Cases and controls were combined for mQTL analysis, as depicted by boxplots

Of the 137 CpGs associated with OCD, 106 were tested with SNPs within 5 kb, and 81% of those (86) were significantly correlated with at least one SNP at an FDR-corrected *p* < 0.05. A total of 2882 SNP-CpG pairs were tested, and 1350 were identified as mQTLs. Select mQTL associations identified in ADHD and OCD can be seen in Fig. 4. For both ADHD- and OCD-associated CpGs sets, the number of CpGs associated with at least one mQTL was significantly enriched (*p* values< 0.001), as compared to 1000 iterations of randomly sampled CpGs (See Methods for greater detail; Supplementary Figure 6).

Finally, we tested each SNP that was significantly associated with an NDD-associated CpG (3283 SNPs correlated with ADHD-associated CpGs and 1150 SNPs correlated with OCD-associated CpGs as identified in the mQTL analysis) against disorder status. No SNPs were significantly associated with OCD after FDR correction; however, 13 SNPs within a 3.5-kb distance and in perfect linkage disequilibrium were associated with ADHD (*p* values<0.05; Supplementary Table 3). These SNPs were intronic to the gene *MAD1L1*, a component of the mitotic spindle-assembly checkpoint. This finding suggests that DNAm may mediate the interaction between ADHD and genomic/genetic variation as this locus.

### mQTL SNPs had skewed *p* values in independent GWAS of ADHD but not OCD

We assessed the summary statistics of two independent GWAS analyses for ADHD and OCD, of European decent, to examine whether mQTL SNPs, i.e., SNPs associated with disorder-associated CpGs, were independently related to disorder status in larger sample sizes.

**Fig. 5:**
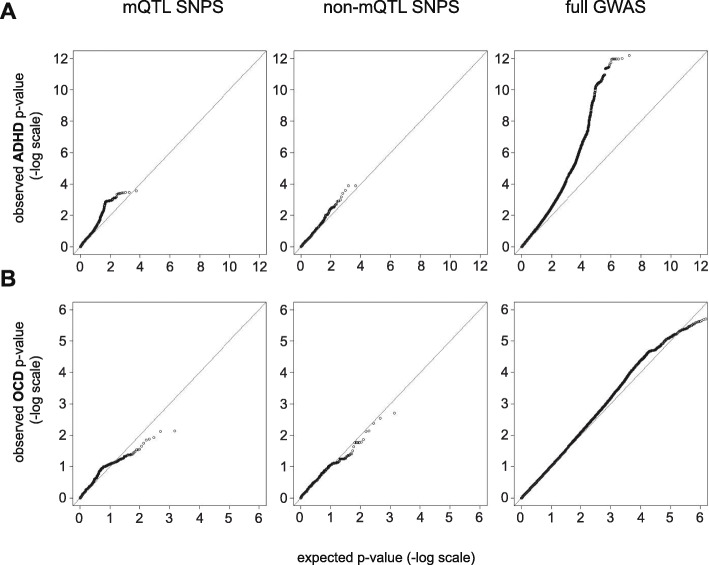
Q-Q plots of independently generated GWAS *p* values in (**a**) ADHD and (**b**) OCD. Plots show *p* value distribution of mQTL SNPs with disorder-associated CpGs (left), non-mQTL SNPs proximal to disorder-associated CpGs (middle), and SNPS from the full GWAS (right)

From the results of Demontis et al. European cohort, we pulled all SNPs that were tested for mQTLs in the ADHD sample; 5064 of 5294 were available, which included 2760 of 2896 mQTL SNPs [19]. We generated Q-Q plots of these mQTL SNPs and the remaining 2304 SNPs that were tested for mQTLs, but not significant (Fig. 5; Table 3). The genomic inflation factor reported for the full GWAS, testing 8,094,094 SNPs, was 1.22. By comparison, the mQTL SNPs (i.e., those associated with ADHD-associated CpGs) had a *λ*=1.47 while in the remaining non-mQTL SNPs *λ*=1.23. This suggested that the discovery of epigenotype-genotype-phenotype relationships was dependent on associations between proximal SNPs and CpGs.

**Table 3:** Comparative genomic inflation factors (λ)

Disorder	subset	*n* CpGs tested	Sample *λ*
ADHD	Full GWAS	8094094	1.22
	mQTL SNPs	2760	1.47
	non-mQTL SNPs^a^	2304	1.23
OCD	Full GWAS	8409516	1.03
	mQTL SNPs	737	1.30
	non-mQTL SNPs^a^	693	1.32

^a^Within 5 KB of disorder-associated with CpG

From the *p* values published in IOCDF-GC and OCGAS (2018) European cohorts, we assessed all SNPs that were tested for mQTLs in the OCD sample in this study in the same manner as described for ADHD [22]. Of 2202 SNPs tested for mQTLs, 1430 were available in the OCD GWAS, which included 737 of 1105 mQTL SNPs and the 693 remaining SNPs that were proximal, but not correlated with OCD-associated CpGs. Unlike the ADHD GWAS analysis, the genomic inflation factor of the OCD mQTL SNPs and non-mQTL SNPs was inflated, *λ* = 1.3 and 1.32, respectively, relative to the full GWAS, *λ*=1.03. However, Q-Q plots showed that “inflation” was limited to *p* values near the mid-point, while more significant *p* values were larger than expected, falling below the line of equality (*y* = *x*).

## Discussion

Genetic and phenotypic heterogeneity of NDDs, including ADHD and OCD, have likely contributed to difficulties in uncovering the molecular etiologies of these disorders. Here, we found that differentially methylated CpGs were more readily identified by epigenome-wide analysis of both ADHD and OCD when groups were reduced to more symptomatic cases. Moreover, the majority of these CpGs were linked to mQTLs, associating with genetic variation at proximal SNPs.

Research into the epigenetic aberrations associated with ASD has provided insight into how epigenetic patterns in blood-derived DNA can be reflective of heterogenous neurodevelopmental phenotypes and how samples may be classified a priori using underlying genetic variation to better define subgroups of ASD [65]. We took a similar approach using phenotype rather than genotype to assess whether a subset of individuals with more endorsed symptoms of ADHD and OCD were more distinct from controls than larger, more heterogeneous cohorts of ADHD and OCD. To account for the possibility of higher comorbidity rates with more severe presentations, participants with multiple diagnoses were excluded. In both disorders, the reduced sets of more symptomatic cases exhibited differential methylation from controls at a greater number of CpGs than the larger cohorts, and they clustered more distinctly from controls. As well, multiple DMRs in both disorders either gained significant CpGs or had larger effect sizes in the subsets of cases with greater clinical severity (Fig. 2). This finding speaks to the potential utility of homogenous group of cases to improve the signal of epigenetic differences from controls.

In both the full OCD cohort and subset selected for greater OCD severity, there was no clear-cut distinction in clustering of cases and controls as visualized on a PCA plot (Fig. 3). Of note, our severity cutoff corresponded to “moderate” OCD on the CY-BOCS and children diagnosed with moderate OCD experience daily interference in their school and social performances; their obsessive thoughts are described as frequent and disturbing, and they can have difficulty controlling or resisting urges to perform compulsions. Nonetheless, in individuals with OCD, DNAm patterning showed greater overlap with that of neurotypical children than the ADHD group (versus controls). Interestingly, in brain imaging studies of NDDs, brain structural connectivity of individuals with ADHD differed more strongly from controls than the structural connectivity of individuals with OCD. Specifically, wide-spread fractional anisotropy, which measures brain tissue characteristics including fibre density and myelination, demonstrates significant reductions in both ASD and ADHD groups as compared to both controls and OCD groups; the OCD group was the most similar to controls, with differences in fractional anisotropy limited to the splenium [66]. Taken together with our findings, these results suggest that ADHD may be a more distinctive condition at the genetic, epigenetic and neurological levels than OCD as compared to neurotypical children.

We found 7 CpGs mapping to 5 genes (*C13orf39*, *C17orf54*, *DNAJC15*, *LLGL2*, *POLS*) that were associated with both ADHD and OCD in the more symptomatic samples (Supplementary Table 2). Additionally, both disorders were associated with altered DNAm at CpGs mapping to *MAD1L1*, *MGC87042*, *PTPRN2*, and *SGK2*; however, the specific associated CpGs were unique to each disorder. Notably, *MAD1L1* has previously been associated with ADHD, as reported in an EWAS of DNAm data measured in saliva samples on the Illumina 450K HumanMethylation array [42]; Wilmot et al. found four CpGs mapping to *MAD1L1* associated with ADHD at a nominal *p* value< 0.05 and Δβ > 2% [42]. In our sample, two CpGs in this gene were associated with ADHD (cg12376829, *p* value< 1.7 × 10^–4^, Δ*β* = − 6.9%; cg17545141, *p* value< 0.044, Δ*β* = 5.7%), and one was associated with OCD (cg03075889, *p* value< 0.037, Δ*β* = 16.1%). In sum, many of our findings suggest that there may be common epigenetic dysregulation across multiple NDDs as has been demonstrated previously for genomic variation.

The *MAD1L1* gene has previously been reported as containing risk variants associated with both bipolar disorder and schizophrenia, in multiple studies, and more recently, with ADHD and anxiety [67–71]. In our analysis, *MAD1L1* contained the only SNPs significantly associated disorder status; 13 SNPs in perfect linkage disequilibrium were associated with ADHD. This gene specifically merits further research with respect to both the genetic and epigenetic variation in associations with NDDs.

Assessing OCD- and ADHD-associated CpGs for associations with genetic variation, we discovered that 81% and 88% of significant sites were linked to mQTLs, respectively. These proportions are relatively high given that depending on tissue and developmental timing, between 20–80% of CpGs are predicted to be mQTL-associated [32–35]. This finding was consistent with previous research into epigenetic correlates of ADHD and schizophrenia, but this is the first demonstration of this finding for OCD [40, 48, 72]. In the context of schizophrenia, mQTLs have been proposed as representing SNPs with a functional annotation [40, 48]. The genetic variation across individuals harboring different SNPs can be associated with a regulatory change that is mediated by a specific DNAm change. Based on this postulation, we tested whether our mQTL SNPs, i.e., SNPs associated with disorder-associated CpGs, were more likely to be associated with disorder status in large, independent GWAS analyses run on ADHD and OCD groups. Using the summary statistics of our mQTL SNPs as compared to the whole genome, we saw a stronger trend towards lower *p* values in ADHD, but not OCD. One interpretation of this finding is that there is a stronger epigenotype-genotype-phenotype correlation in ADHD than OCD and therefore, incorporating DNAm into ADHD genetic research may be particularly fruitful as it has been in schizophrenia research. Although the OCD GWAS we used was the largest study to date, it is still likely underpowered, with no reported SNPs meeting genome-wide significance. As such, we cannot definitively say that DNAm would not be informative in future OCD GWAS analyses.

Our findings were limited by common issues that affect epigenetic research in NDDs. DNAm is strongly associated with tissue/cell type and here, we have analyzed saliva DNAm in cases and controls rather than brain, which is arguably the tissue of interest. As correlations between saliva and brain tissue are limited, we hesitate to interpret potential effects of these DNAm patterns on brain pathophysiology and how they relate to ADHD or OCD etiology. Nonetheless, DNAm studies in accessible tissues, such as saliva and peripheral blood, have contributed to the understanding of the pathophysiology of complex diseases, gene-environment interactions, and effects of prenatal exposures, all of which are pertinent to the study of neurodevelopmental disorders [48, 60, 73, 74]. As well, such accessible, quantitative measures may prove useful as molecular markers of each disorder, potentially prior to clinical presentation and predictive of later behavioral outcomes.

Furthermore, our study sample was small and likely underpowered, especially given that common genetic variants are believed to have small contributions to disorder risk, and it is plausible that similar effects are seen in epigenetics. However, based on our findings, we argue that the reduced power of selecting a subset of cases may be offset by the increased effect size, as seen in our DMRs. Finally, as both ADHD and OCD are believed to represent extremes of quantitative traits, it is likely that our control samples reflect the normative degree of heterogeneity seen in SWAN and CY-BOCS measures [56]. As such, our findings were likely affected by the ranges of non-syndromal ADHD and OCD traits in the control group. Future studies would ideally measure ADHD or OCD in both cases and controls to have a better understanding of the range of phenotypic variability and overlap in each group prior to assessing DNAm.

## Conclusions

The enrichment of mQTLs in NDD-associated CpGs sites, presented here and in previous research studies, highlights the utility of DNAm as both an asset to genetic NDD research and a potential biomarker in itself. The DNAm patterns in ADHD and OCD provide evidence of potential epigenetic biomarkers mirroring the phenotypic heterogeneity of these NDDs. Across all NDD research, it is plausible that reducing NDD cohorts to more homogenous subgroups may be a useful method in uncovering stronger molecular correlates as we have shown here.

## Supplementary information

**Additional file 1: Supplementary Figure 1.** Principal components 1 and 2 from principal component analysis (PCA) of our samples and samples from phase 3 of the 1000 Genomes project. PCA was calculated from ancestry informative markers (AIM). Plotted samples included our control, ADHD and OCD samples (black) and 1000 Genomes project samples grouped into the following ancestries: African (AFR), Americas (AMR), East Asian (EAS), European (EUR), and South Asian (SAS). Samples represented by black a “X” were identified as outliers (see Methods) and were removed prior to analysis.

**Additional file 2: Supplementary Figure 2.** Distributions of predicted buccal epithelial (buccal) and granulocyte (gran) proportions in “more symptomatic” ADHD subset (n = 15) versus all controls (A; n = 35) and subset of controls (B; n = 27). Underlying cell proportions of saliva samples were predicted from DNAm data using methods described in Smith *et al.* (2015). **(A)** Subset of ADHD samples selected based on ≥6 SWAN symptoms (n = 15) with visibly different cell proportions than corresponding controls (although means did not differ significantly, *p*-values >0.05). **(B)** Same subset of ADHD samples and selected controls, chosen to better balance buccal and granulocyte proportion.

**Additional file 3: Supplementary Figure 3.** Age distribution of cases and controls in each comparison. Ages of participants used in full OCD cohort vs. controls, (top left) full ADHD cohort vs. controls (bottom left), subset of more symptomatic OCD cases with CYBOCS scores ≥18 vs. controls (top right), and subset of more symptomatic ADHD cases with SWAN scores ≥6 vs. controls (bottom right). Asterisk denotes significant difference in mean ages between groups (*p*-value<0.05).

**Additional file 4: Supplementary Figure 4.** Silhouette plots generated on betas values using Manhattan distance, using the same samples and CpGs as displayed in Figure 3a. **(A)** all OCD samples and controls (n = 113, CpGs = 82); **(B)** more symptomatic OCD samples and controls (n = 82, CpGs = 137); **(C)** all ADHD samples and controls (n = 57, CpGs = 188); **(D)** more symptomatic ADHD samples and controls (n = 42, CpGs = 299).

**Additional file 5: Supplementary Figure 5.** More symptomatic cases cluster more distinctly from controls using CpGs identified in the full cohorts, than full cohorts using CpGs identified in the subsets. PCAs were run on subsets using NDD-associated CpGs identified in full cohorts, and on full cohorts using NDD-associated CpGs identified in subsets. Samples sizes and number of CpGs input into PCA shown in bottom, righthand corner of each facet.

**Additional file 6: Supplementary Figure 6.** Random distribution of CpGs associated with at least one mQTL from sets of 299 CpGs (left) and 137 CpGs (right). Sets of CpGs randomly sampled from preprocessed EPIC array data 1000 times were correlated against SNPs to generate distributions of expected numbers of mQTL-associated CpGs identified (top) and expected proportions of mQTL-associated CpGs. Red lines represent numbers of ADHD- or OCD-associated CpGs found to be associated with at least one mQTL.

**Additional file 7: Supplementary Tables S1, Table S2, Table S3.**

## Data Availability

The microarray data will be made publicly available in the GEO repository upon publication.

## Abbreviations

ADHD: Attention-deficit/hyperactivity disorder
BEC: Buccal epithelial cell
DMR: Differentially methylated region
DNAm: DNA methylation
EWAS: Epigenome-wide association study
GWAS: Genome-wide association study
mQTLs: Methylation quantitative risk loci
NDD: Neurodevelopmental disorders
OCD: Obsessive-compulsive disorder
PCA: Principal component analysis
SNP: Single nucleotide polymorphism
