# Supplementary figures and images for: Obsessive-compulsive disorder and attention-deficit/hyperactivity disorder: distinct associations with DNA methylation and genetic variation
Source: J Neurodev Disord. 2020 Aug 16;12:23. doi: 10.1186/s11689-020-09324-3 (PMC7429807; doi:10.1186/s11689-020-09324-3)

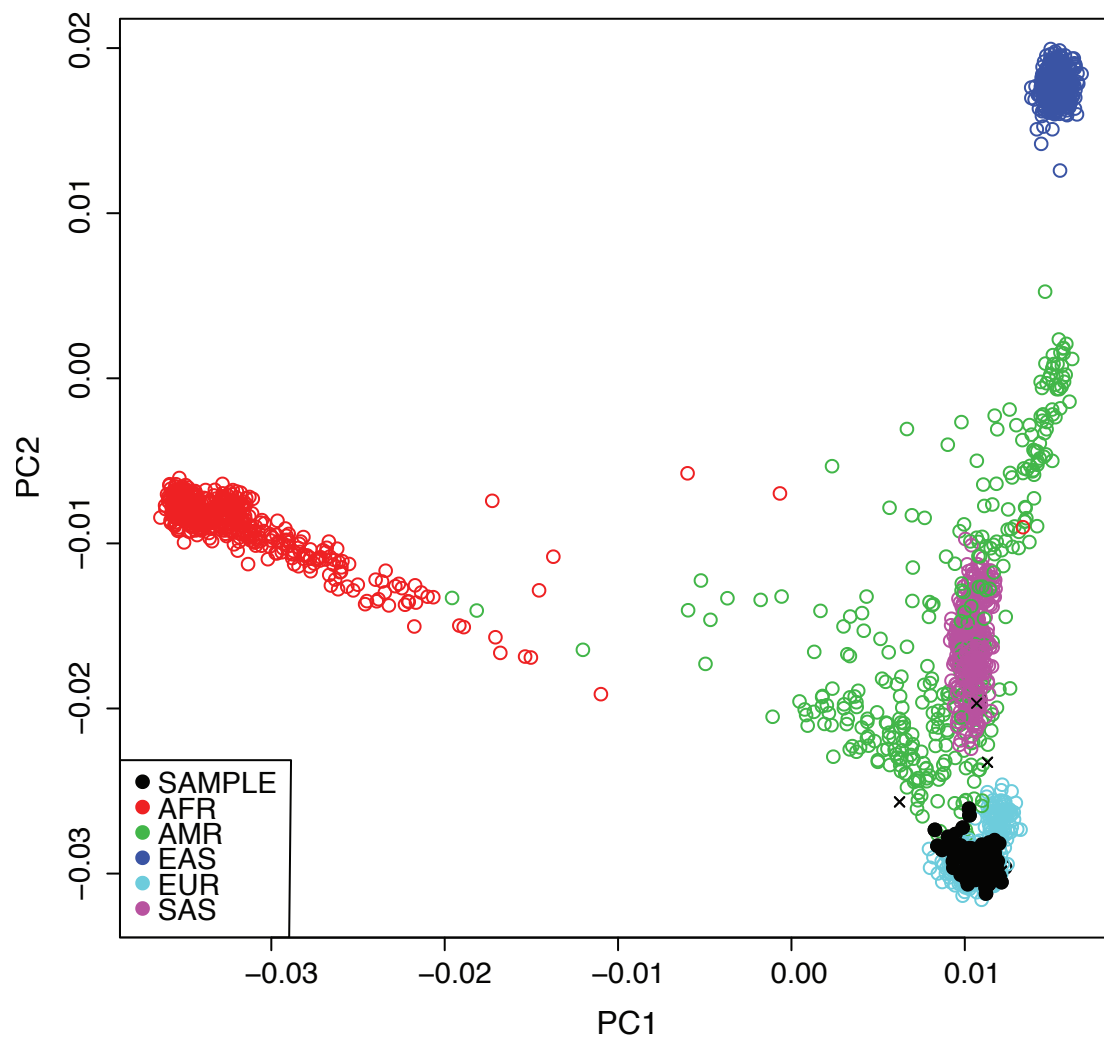

Supplement: Supplementary file 1 — Additional file 1: Supplementary Figure 1. Principal components 1 and 2 from principal component analysis (PCA) of our samples and samples from phase 3 of the 1000 Genomes project. PCA was calculated from ancestry informative markers (AIM). Plotted samples included our control, ADHD and OCD samples (black) and 1000 Genomes project samples grouped into the following ancestries: African (AFR), Americas (AMR), East Asian (EAS), European (EUR), and South Asian (SAS). Samples represented by black a “X” were identified as outliers (see Methods) and were removed prior to analysis. [file 11689_2020_9324_MOESM1_ESM.pdf]

**A**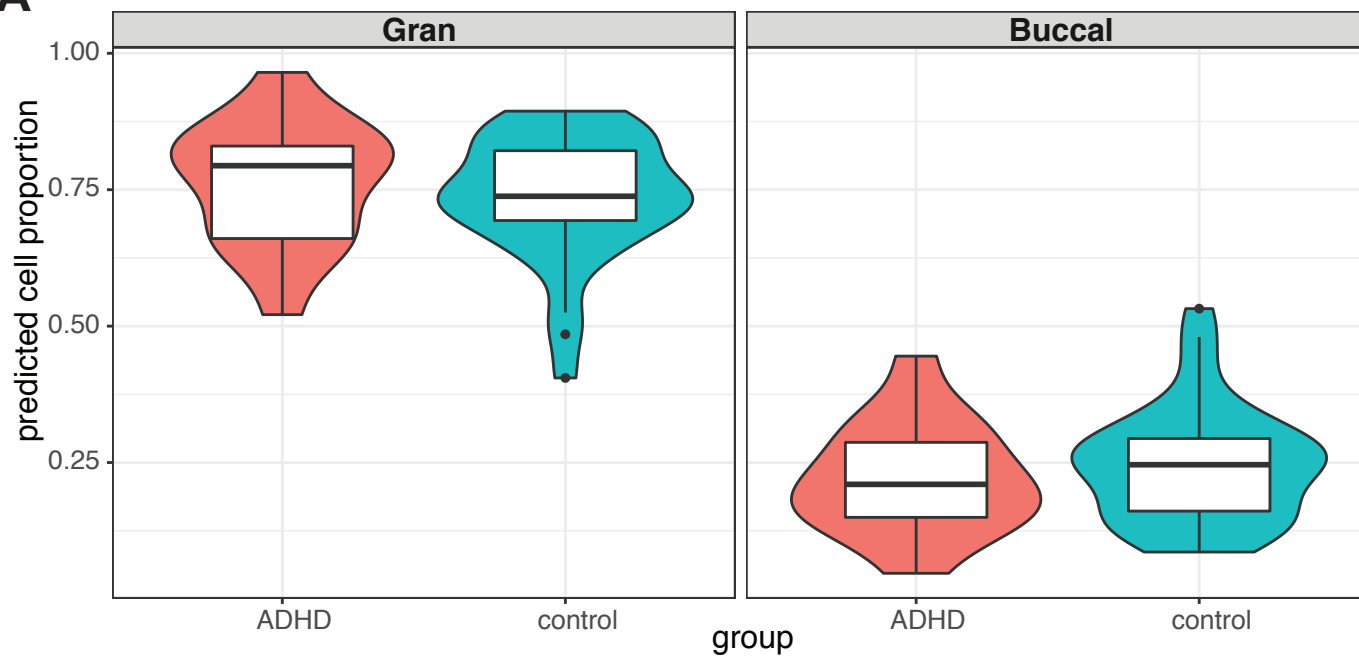**B**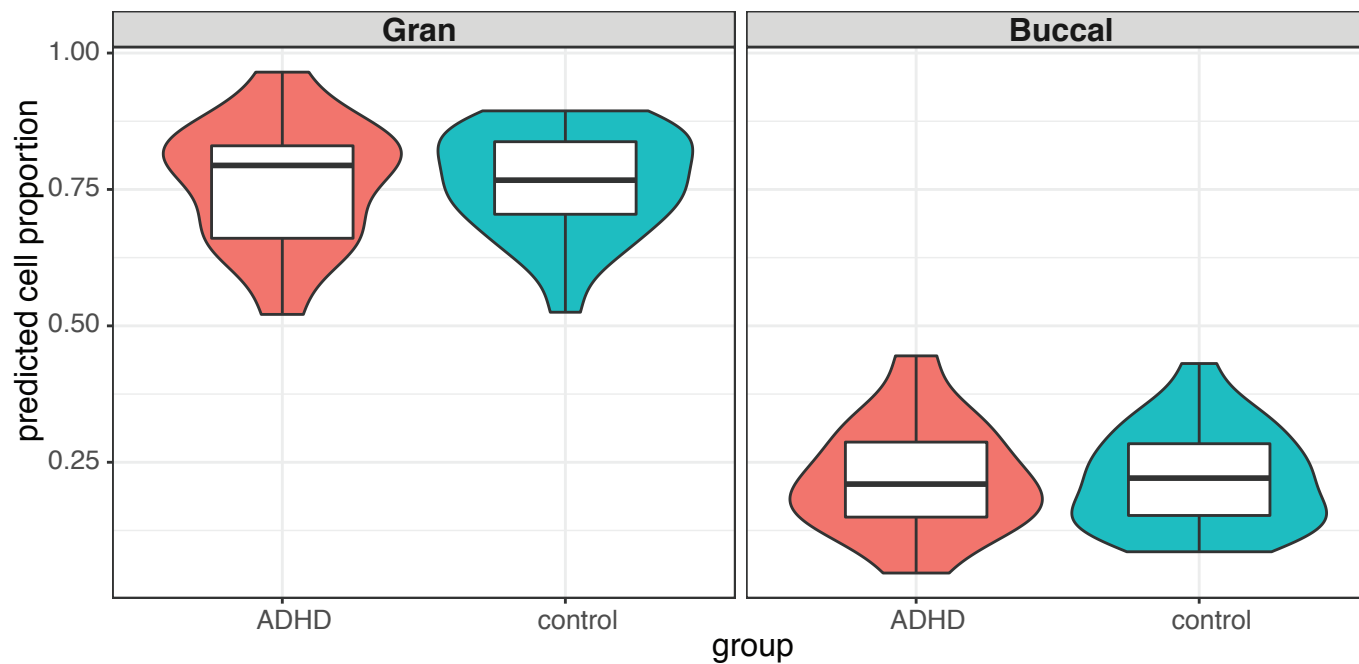

Supplement: Supplementary file 2 — Additional file 2: Supplementary Figure 2. Distributions of predicted buccal epithelial (buccal) and granulocyte (gran) proportions in “more symptomatic” ADHD subset (n = 15) versus all controls (A; n = 35) and subset of controls (B; n = 27). Underlying cell proportions of saliva samples were predicted from DNAm data using methods described in Smith et al. (2015). (A) Subset of ADHD samples selected based on ≥6 SWAN symptoms (n = 15) with visibly different cell proportions than corresponding controls (although means did not differ significantly, p-values >0.05). (B) Same subset of ADHD samples and selected controls, chosen to better balance buccal and granulocyte proportion. [file 11689_2020_9324_MOESM2_ESM.pdf]

All samples

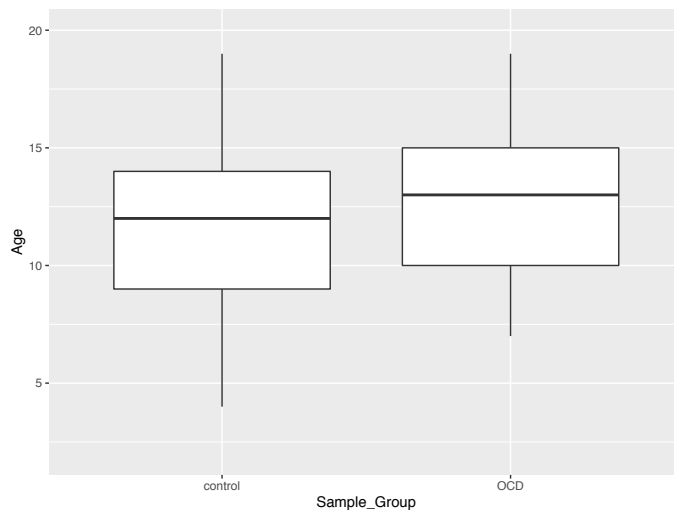

Subset of cases  
(SWAN  $\geq 6$  or CY-BOCS  $\geq 18$ )

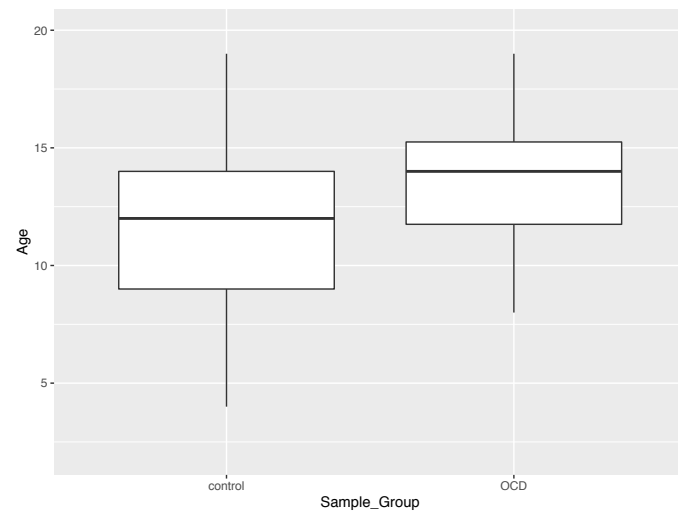

ADHD vs. controls

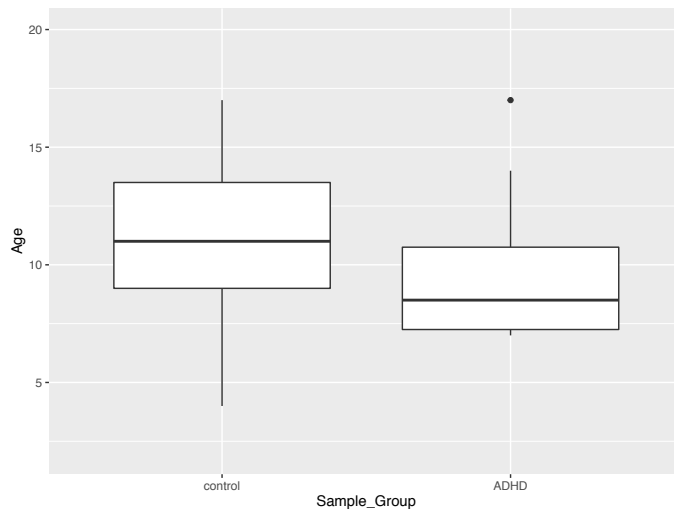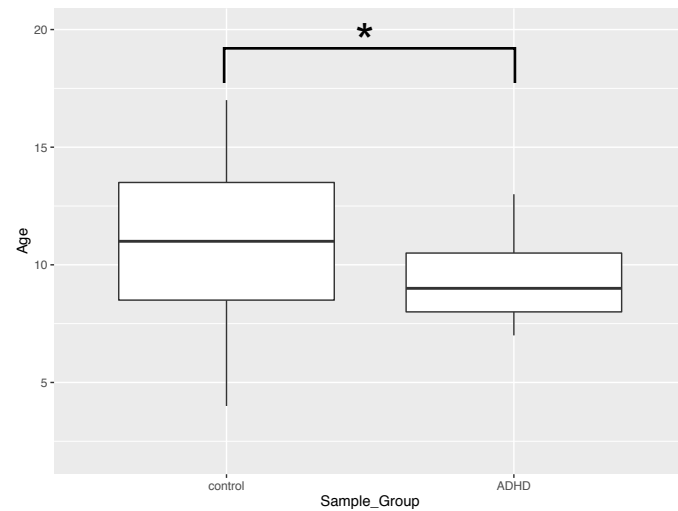

Supplement: Supplementary file 3 — Additional file 3: Supplementary Figure 3. Age distribution of cases and controls in each comparison. Ages of participants used in full OCD cohort vs. controls, (top left) full ADHD cohort vs. controls (bottom left), subset of more symptomatic OCD cases with CYBOCS scores ≥18 vs. controls (top right), and subset of more symptomatic ADHD cases with SWAN scores ≥6 vs. controls (bottom right). Asterisk denotes significant difference in mean ages between groups (p-value<0.05). [file 11689_2020_9324_MOESM3_ESM.pdf]

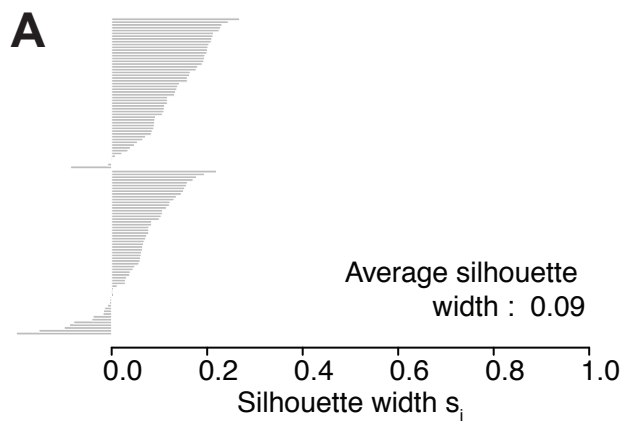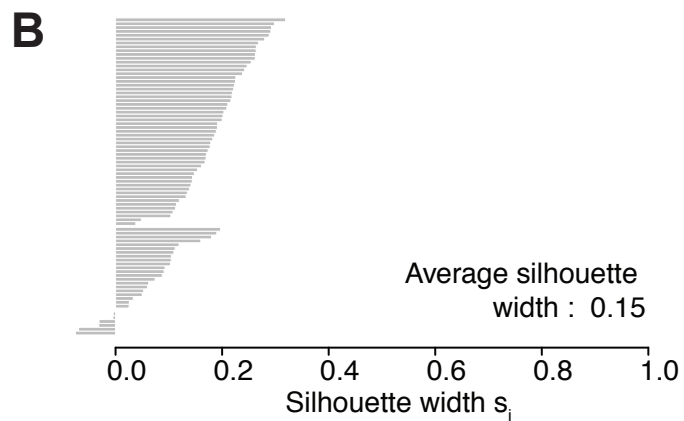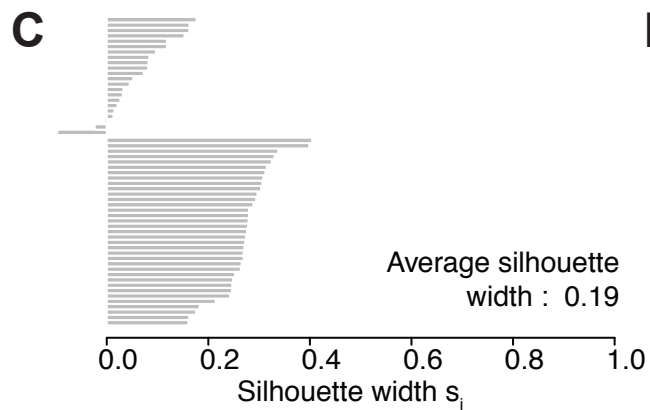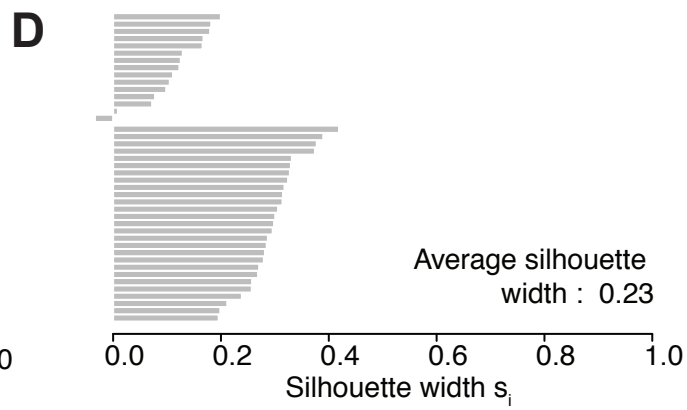

Supplement: Supplementary file 4 — Additional file 4: Supplementary Figure 4. Silhouette plots generated on betas values using Manhattan distance, using the same samples and CpGs as displayed in Figure 3a. (A) all OCD samples and controls (n = 113, CpGs = 82); (B) more symptomatic OCD samples and controls (n = 82, CpGs = 137); (C) all ADHD samples and controls (n = 57, CpGs = 188); (D) more symptomatic ADHD samples and controls (n = 42, CpGs = 299). [file 11689_2020_9324_MOESM4_ESM.pdf]

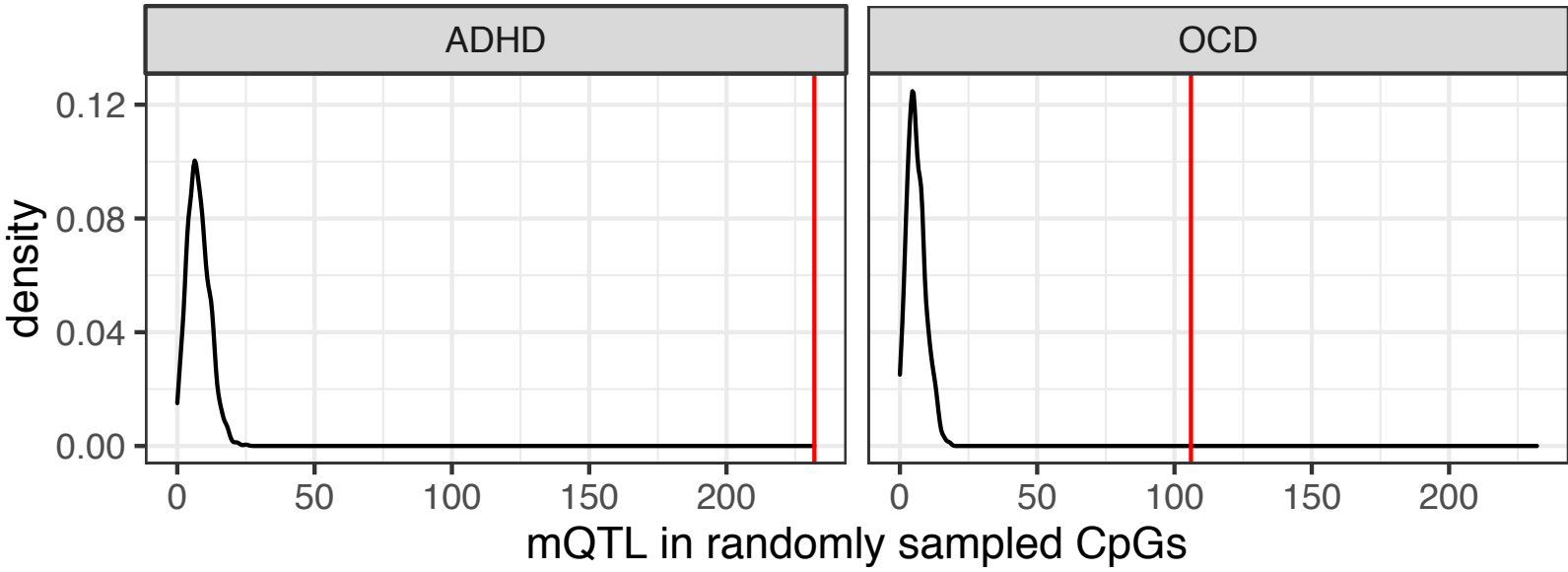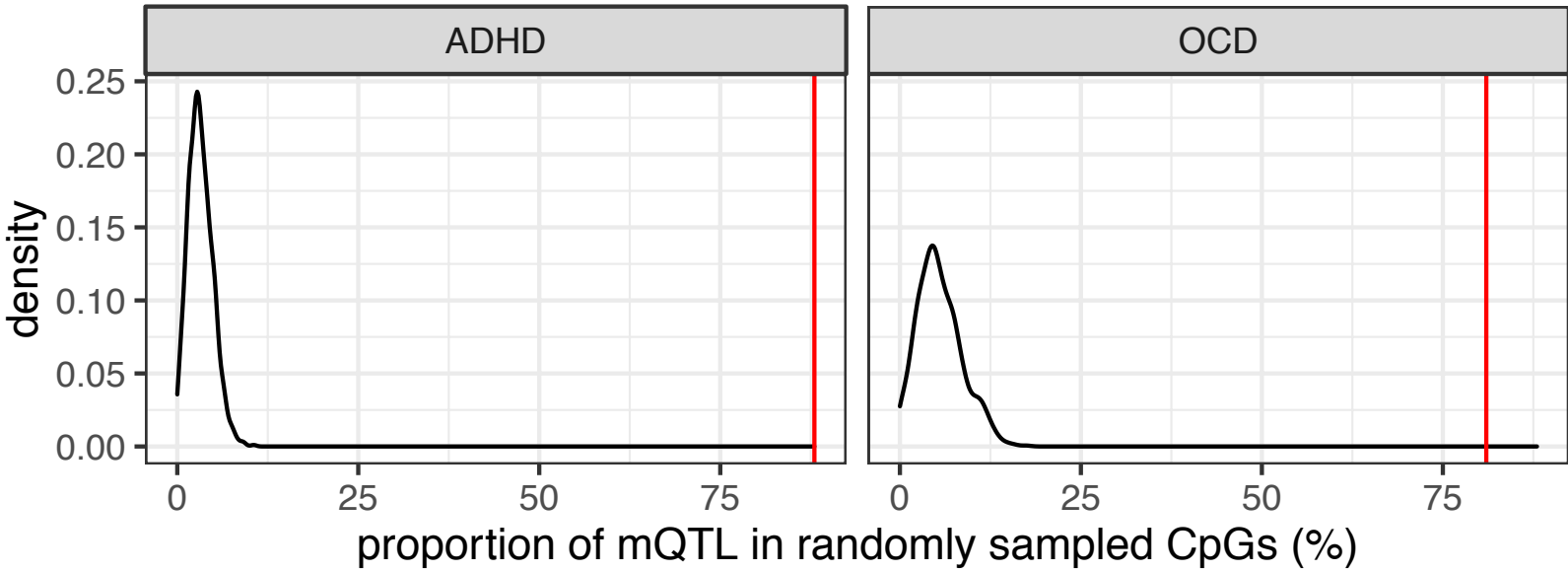

Supplement: Supplementary file 6 — Additional file 6: Supplementary Figure 6. Random distribution of CpGs associated with at least one mQTL from sets of 299 CpGs (left) and 137 CpGs (right). Sets of CpGs randomly sampled from preprocessed EPIC array data 1000 times were correlated against SNPs to generate distributions of expected numbers of mQTL-associated CpGs identified (top) and expected proportions of mQTL-associated CpGs. Red lines represent numbers of ADHD- or OCD-associated CpGs found to be associated with at least one mQTL. [file 11689_2020_9324_MOESM6_ESM.pdf]
