# Supplementary material for: Obsessive-compulsive disorder and attention-deficit/hyperactivity disorder: distinct associations with DNA methylation and genetic variation
Source: J Neurodev Disord. 2020 Aug 16;12:23. doi: 10.1186/s11689-020-09324-3 (PMC7429807; doi:10.1186/s11689-020-09324-3)
Supplement: Supplementary file 5 — Additional file 5: Supplementary Figure 5. More symptomatic cases cluster more distinctly from controls using CpGs identified in the full cohorts, than full cohorts using CpGs identified in the subsets. PCAs were run on subsets using NDD-associated CpGs identified in full cohorts, and on full cohorts using NDD-associated CpGs identified in subsets. Samples sizes and number of CpGs input into PCA shown in bottom, righthand corner of each facet. [file 11689_2020_9324_MOESM5_ESM.pdf]

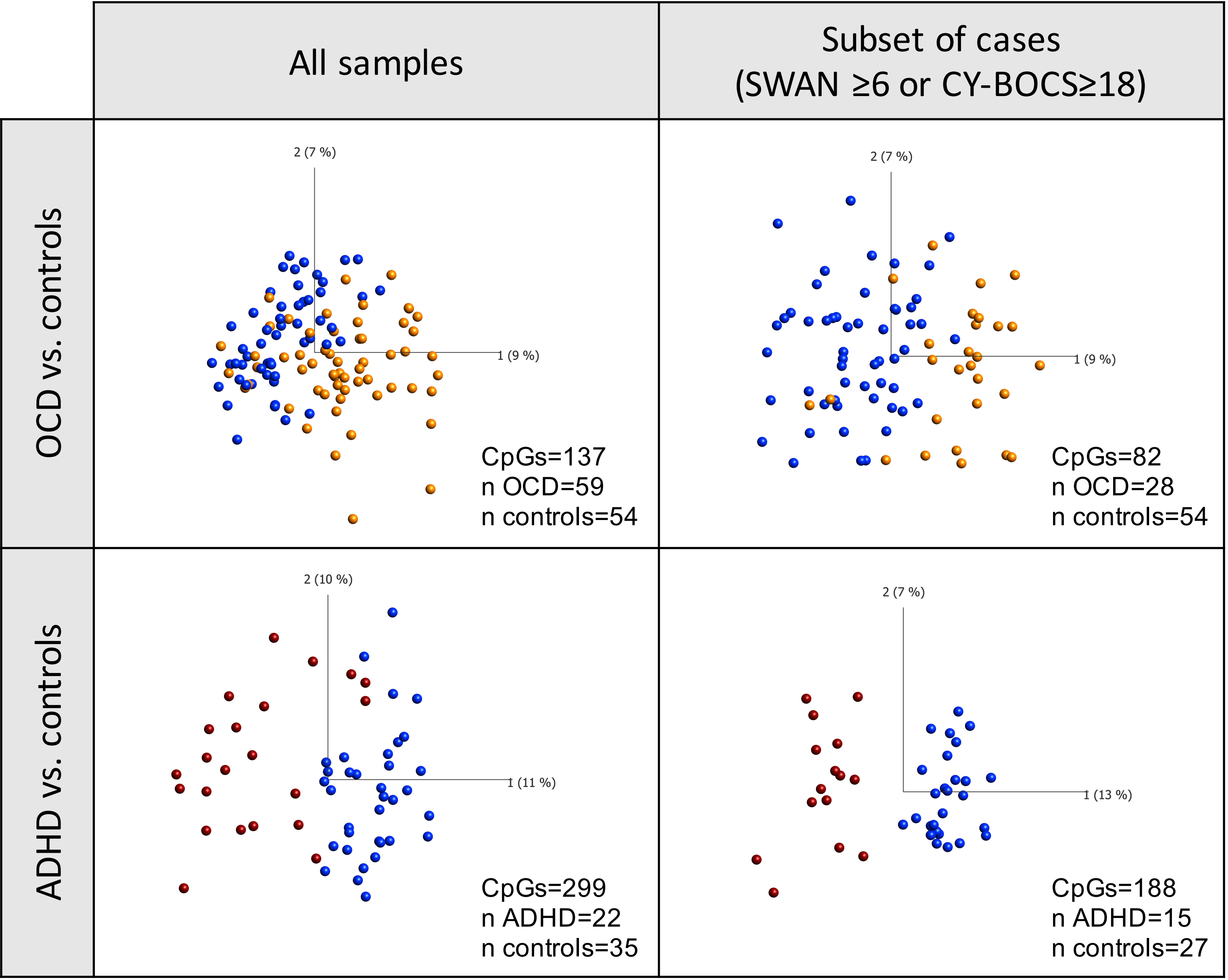
All samples
Subset of cases (SWAN ≥6 or CY-BOCS≥18)
OCD vs. controls
ADHD vs. controls
2 (7 %)
1 (9 %)
CpGs=137
n OCD=59
n controls=54
2 (7 %)
1 (9 %)
CpGs=82
n OCD=28
n controls=54
2 (10 %)
1 (11 %)
CpGs=299
n ADHD=22
n controls=35
2 (7 %)
1 (13 %)
CpGs=188
n ADHD=15
n controls=27
